# Supplementary material for: Effects of health education intervention on menstrual hygiene knowledge and practices among the adolescent girls of Pokhara Metropolitan, Nepal
Source: PLoS One. 2024 Sep 18;19(9):e0291884. doi: 10.1371/journal.pone.0291884 (PMC11410211; doi:10.1371/journal.pone.0291884)
Supplement: Attachment — Submitted filename: Rebuttal Letter.docx [file pone.0291884.s002.docx]

**Rebuttal Letter**

**Dear Academic Editor and Reviewer,**

We are honored and delighted to have been given the opportunity for submitting a revised version of the manuscript that addresses the points raised during the review process**.**

In face of PLOS ONE's decision for the manuscript ("Decision: Revision required **[PONE-D-23-28751]-[EMID: b2f3fa8ec33e93d6]**. We appreciate the insightful feedback provided by the editor and reviewers, which helped us to refine the revised version of the manuscript that was submitted.

All the comments were considered providing the following changes to the points raised by the academic editor and reviewers.

**Upon resubmission, please provide the following:**
 
The name of the colleague or the details of the professional service that edited your manuscript:

**Response:** The identity of the associate that proofread this manuscripts is mentioned.

A copy of your manuscript showing your changes by either highlighting them or using track changes (uploaded as a *supporting information* file).

**Response:** A copy of manuscript showing changes was done using track changes and it is uploaded as a *supporting information* file.

A clean copy of the edited manuscript (uploaded as the new *manuscript* file)”.

**Response:** A clean copy of the edited manuscript has been uploaded as the new *manuscript* file)”.

Note that the grant information you provided in the ‘Funding Information’ and ‘Financial Disclosure’ sections do not match.

**Response:** My apologies, it has been now been corrected. Thank you

Ensure that to provide the correct grant numbers for the awards you received for your study in the ‘Funding Information’ section.

**Response**: Apologies for the oversight. The grant number was 438 with amount, NPR 40,000 (USD 300), which was very small amount which was only used for the data collection.

Your ethics statement should only appear in the methods section of your manuscript. If your ethics statement is written in any section besides the Methods, please move it to the Methods section and delete it from any other section.

**Response:** Thank you for your note. An ethical statement is now written in the end of the methods section.

All Figure in the supporting information in your submission contain copyrighted images. We require you to either (1) present written permission from the copyright holder to publish these figures specifically under the CC BY 4.0 license, or (2) remove the figures from your submission:

**Response:** Thank you for asking a copy right permission letter. Unfortunately we do not have written permission of images. Therefore, we would like to remove all the figures if it is unethical. However, majority of photos were taken from own mobile phone. Therefore we have decided to remove those images.

Reviewer’s comments:

Reviewer #1

1. Abstract should be limited to the 300 words, please follow the author's guideline of the journal.

**Response:** Thank you for your comments. The revised manuscript abstract is limited to the required words based on journal author's guideline. Now the abstract word count is 300 excluding keywords.

2. It is better to put a keywords in alphabetical order.

**Response:** Thank you for your guidance. Keywords are now arranged in alphabetical order in the revised manuscript.

3. Please include the line number in manuscript file, it would be easier to comment and author would easily addressed those comments.

**Response:** Thank you for your suggestion. We have now created line numbers in the revised manuscript.

4. Please complete the sentence appropriately, some of the lines are difficult to understand and some are incomplete.

**Response:** Thank you for your valuable feedback. All the sentences are checked appropriately and completed in understandable language in revised manuscript.

5. While citing in the text, please include the author's name or where the study is done instead of writing most of the studies, some studies.

**Response:** Thank you for making us more sincere. Now, we have added original citation in the text and the study location is now included instead of just writing most of the studies or some studies.

6. Would you please justify choosing the study site, as only government schools were chosen and only two schools were taken.

**Response:** We appreciate your question. Thank you. We would like to give some insight on it. The previous studies conducted at schools especially among South Asian and Sub-Saharan African showed, girls who attend government schools were more likely to have poor menstrual hygiene because of their low level of socioeconomic status [19].The selection of government schools as the study site was deliberate and aimed to ensure a representative sample of adolescent girls from a specific socioeconomic background who might be most in need of support. Government schools were chosen for their accessibility and cooperation in research activities. Additionally, two schools were selected for in-depth investigation within the constraints of available resources, time and logistical challenges associated with executing the intervention. **(Reference is cited in manuscript).**

7. Would you please give a reference regarding the age of adolescents.

**Response:** Adolescent’s age group age between 10-15 is crucial period where adolescents girls had to deal with fear, stigma, discomfort, superstition and many more during menstruation especially in poor countries like Nepal. In this period right information and practice should be promoted so that their menstrual health of those adolescents is maintained for long term. The research conducted on Nepal, Egypt, Kenya, South Africa, India have recommended for conducting menstrual hygiene and health interventions as early as possible so as to equip adolescent’s girls with scientific knowledge and hygienic practices regarding menstruation [16,21–25]. Average age of menarche among school going adolescents was between 10-15 years in this study and study conducted by these studies [5,28, 35–37]. Even though national school curriculum on menstrual hygiene in textbooks have been updated from grade 4-12 [11], still more than half adolescents have inadequate information about menstruation and only one in ten are practicing good menstrual hygiene[12].The research conducted in developing nations such as Nepal, India, and Nigeria [5, 28, 35–37] revealed that the mean age of menarche among school-going adolescents falls within the range of 10 to 15 years. Research conducted by Fetohy EM in Egypt and Chang and Chen in the Hualien Region has highlighted the importance of expanding menstrual hygiene education to elementary, preparatory, and other secondary schools, especially during crucial transitional periods such as menarche. Furthermore, their studies have demonstrated the effectiveness of educational initiatives in these population in enhancing menstrual health [21,27]. Therefore, this age group was selected as the target population for the study**. (Reference is cited in manuscript).**

8. Have you gave any educational sessions to the non-intervention group after the study survey? As schools were randomly assigned as interventional and non-interventional groups.

**Response:** Yes. We have conducted educational sessions like video playing, PowerPoint presentation, group discussion, simulation, handouts distribution in non-intervention group as well after the study survey.

9. It is very unclear that why the participants having irregular periods were excluded from the study, would you like to give a clarification regarding this?

**Response:** Thank you for raising this issue. Participants having irregular periods were excluded from the study especially in data collection period but not while conducting sessions. It was done to ensure a more homogeneous sample, allowing for a clearer assessment of the intervention's effects on menstrual practices among individuals with regular cycle. Given the short duration of the intervention (one month), participants with irregular menstrual cycles may not experience the full benefits of the intervention or may have difficulty adhering to the study practical aspect which was done through simulation.

10. Would you like to explain this sentence, 'A systematic review of existing scientific articles, official reports and policies on menstrual hygiene was done' and how it was done?

Response: Thank you for the note. We apologize, it was intended to say that performing a literature review prior to conducting a study, but the way it was written conveyed an inaccurate message, thus it has been fixed now in the revised manuscript.

11. Would you please mention the version of SPSS and license number as data was transformed into SPSS.

**Response:** SPSS version 21 was used. We have included this information in our revised manuscript. Thank you.

12. Would you please mention the reference of categorization of observed practice level?

**Response:** Our literature review did not find any published article that provided guidance on categorizing ‘observed practice level’. Therefore, we used median as cut off point to categorize as poor and good practice as data were not normally distributed. If it is unsuitable to categorize observed practice level this way it can be discarded.

13. In table number 3, 4 and, 5 statistical methods were not mentioned.

**Response:** Statistical methods now has been mentioned in table number 3, 4 and 5 and below table. We have also included this information in statistical section and have indicated respective table number. In table 3, Chi square and Fisher’s exact test was used to assess difference between interventional and non-interventional group regarding socio-demographic information. For inferential statistics Wilcoxon signed rank test was used.

14. It would be better if you could shows the associations between knowledge and practice of the participants.

**Response:** We appreciate your question. Thank You for insight. The primary aim of this paper was to assess the effects of a health education intervention on menstrual hygiene knowledge and practices among adolescent girls, rather than to explore the associations. Because sample sizes are small, it may lack the ability to detect true associations between groups and may not provide meaningful or reliable results. We will consider examining the association between knowledge and practice of the participant’s in future interventional studies with larger sample sizes.

15. As the sample size is very small, results could not be generalized to overall Pokhara municipality.

**Response:** Thank you for this feedback. We have included this as a limitation of our study.

16. In discussion, how you compare with median age of the study with average age of participants of other studies?

**Response:** In my study, the data was not normal. So, non-parametric test was used and median age was used. In similar kind of studies, the data was parametric and hence, mean age was used. As, my age categorization was very narrow between 10-15 years there was not much difference in age comparison with other study that have used mean. As aim of the study was to assess the effects of health education intervention on menstrual hygiene knowledge and practice on menstrual hygiene. Age comparison could be given less important if needed based on your feedback comparison with average will be discarded.

17. Would you please write the conclusions of the study rather than writing the recommendations?

**Response:** Thank you for your valuable comment. Conclusions of the study is now revised based on your suggestions. Now it has been written with key findings of the study.

Reviewer #2:

1. The language of the manuscript is poor and will require extensive improvements to make it readable. Repeated words, poor grammar, and conflicted sentence structure make it a difficult read.

**Response:** Thank you for your insightful comment. The language review is now done by an academic and professional reviewer. We revised manuscript thoroughly and rechecked for repeated words, poor grammar and conflicted sentence structure.

2. The Abstract is lengthy and the Methods do not describe the intervention adequately. Major restructuring is required. Consider this sentence as a reflection for the language in the entire manuscript – “A pre-test was done using structured questionnaire and observation checklist for data collection on menstrual hygiene followed by health education interventional sessions in the intervention groups and no any session were conducted in non-intervention group.”

**Response:** Thank you for the comment. Due to constraints on word count, the methods section limited detailed description of the intervention. Therefore, detail information regarding the intervention has been included in the materials and methods section. Now the abstract is limited to 300 words in consistency with first reviewer’s comment.

3. The sample size of 80 makes it a pilot study. The tools utilized were piloted in 10% of the total sample (n=8). Difficult to make statements about validity and reliability with 8 participants.

**Response:** Thank you for the insightful question. Although our health education intervention results indicate promising outcomes for the studied population, it's crucial to acknowledge the limits of our research, especially the small sample size. Piloting tools on only 10% of the sample (n=8) may indeed limit the ability to make definitive statements about validity and reliability. It's important to acknowledge this limitation and consider it when interpreting the results. A pilot study was conducted at one basic school, involving the distribution of questionnaires. The questionnaire were pretested to check the reliability and validity. The aim was to assess the feasibility of the planned research and identify any issues with the questionnaire. Few corrections came from the pilot study, and later all questions were found to be clear.

4. Not giving any intervention to the control group despite knowing the problem there, raises ethical concerns. Educational intervention in one group, educational + practical + discussion in another group would have been ethically suited. Have the authors at least provided any intervention to the control group after the end of the study?

Response: Thank you for your comments. Health education session was conducted in control group after posttest in both group using PowerPoint, video, simulation, handouts distribution, group discussion knowing the presence of menstrual hygiene issues in non-intervention group as well.

5. Only a single 2 hr 10 min intervention in one month for the group seems inadequate. What have the authors done to avoid socially desirable responses?

**Response:** We are grateful for your concerns. We focused on using resources efficiently, involving PowerPoint presentations, videos, group discussions and simulations which were more focused on practical aspect that convey powerful message. This approach aimed to improve both understanding and practice of menstrual hygiene within short period of time, avoiding the need for lengthy and repetitive sessions. Designing of intervention was drafted by first author, then discussion was done with expert and supervisors then was finalized with the help of supervisors. Data were collected by fourth author and first authors facilitated in collection who were taken training from the university prior data collection. Concurrent supervision was done by the supervisors. All data were kept securely and privacy was maintain in the password protected laptop.

Author have carried out following activity to avoid socially desirable responses

- Person who gave the intervention was different from one who administered the questionnaire and was given once because participants in repetitive educational interventions may feel pressure to conform to perceived social norms or expectations, leading to social desirability bias. They may provide responses that they believe are socially acceptable or desirable, rather than expressing their genuine opinions or behaviors leading to biased conclusions. .
- It was anonymous surveys to encourage participants to provide honest responses without fear of judgment or societal expectations.

6. .It is recommended that the authors concise the manuscript to about 1000 words (200 Intro, 250 Methods, 250 Results and 300 Discussion) and submit it to another journal as a Correspondence / Letter to Editor / Brief Communication.

**Response:** We thank you for the reviewer for their intellectual input and also an alternative to our current submission. We have responded to all the concerns raised by the reviewers and the editor. We have edited our manuscript and reduced its word count. We hope that this meets the expectation of the reviewers and the academic editor.
