# Supplementary material for: Effects of health education intervention on menstrual hygiene knowledge and practices among the adolescent girls of Pokhara Metropolitan, Nepal
Source: PLoS One. 2024 Sep 18;19(9):e0291884. doi: 10.1371/journal.pone.0291884 (PMC11410211; doi:10.1371/journal.pone.0291884)
Supplement: Attachment — Submitted filename: Response to Reviewers.docx [file pone.0291884.s003.docx]

**Rebuttal letter**

**Dear Academic Editor and Reviewer,**

We are honored and delighted to have been given the opportunity to submit final revised version of the manuscript that addresses the points raised during the review process**.**

In the face of PLOS ONE's decision for the manuscript ("Decision: Revision required **[PONE-D-23-28751R1]-[EMID: c7e1a1d379c8c8d6]**. We appreciate the insightful feedback provided by the editor and reviewers, which helped us refine the revised version of the submitted manuscript.

All the comments were considered providing the following changes to the points raised by the academic editor and reviewers.

**Upon resubmission, we have provided the following:**

**A rebuttal letter that responds to each point raised by the academic editor and reviewer(s). You should upload this letter as a separate file labeled 'Response to Reviewers'.**

**Response:** A rebuttal letter that responds to each point raised by the academic editor and reviewer(s) has been uploaded as a separate file labeled 'Response to Reviewers'.

**A marked-up copy of your manuscript that highlights changes made to the original version. You should upload this as a separate file labeled 'Revised Manuscript with Track Changes'.**

**Response:** A marked-up copy of our manuscript that highlights changes made to the original version has been uploaded as a separate file labeled 'Revised Manuscript with Track Changes'.

**An unmarked version of your revised paper without tracked changes. You should upload this as a separate file labeled 'Manuscript'.**

**Response:** An unmarked version of our revised paper without tracked changes. This has been uploaded as a separate file labeled 'Manuscript'.

**Regarding the financial disclosure, no changes are required at this time it was a very small amount and was utilized only for data collection.**

**Journal Requirements:**

**Please review your reference list to ensure that it is complete and correct. If you have cited papers that have been retracted, please include the rationale for doing so in the manuscript text, or remove these references and replace them with relevant current references. Any changes to the reference list should be mentioned in the rebuttal letter that accompanies your revised manuscript. If you need to cite a retracted article, indicate the article’s retracted status in the References list and also include a citation and full reference for the retraction notice.**

**Response:** All the reference lists were reviewed to ensure completeness and correctness. The cited papers that were retracted for example reference list number 18 and 53 were removed. Reference list numbers 6,10,15,16,20,22,24,30,37,40,41,51, was checked for completeness. Reference lists numbers 6 and 15 were made as one after checking for completeness and correctness as both references were from the same paper.

**Reviewer’s comments:**

1. **Authors have described how the schools were selected. A justification on how the participants were selected in the classrooms is required. How was the sample size achieved? What did the authors do if the total number of eligible participants went beyond 40? Were some participants rejected just because the sample size of 40 was achieved? Why didn't the authors think of having a similar proportion of participants from class 6 to 8?**

**Response:** Thank you for the insights. The sample size of 40 participants was achieved based on the previous study which we have cited in the materials and methods section (sample size calculation part) in the manuscript. Participants were not rejected simply to achieve a sample size of 40 rather every effort was made to include participants who met eligible criteria like age, attendance of menarche, obtaining written informed assent, prioritizing inclusivity and ethical considerations within the predetermined sample size.

First of all eligible participants were listed from each grade then samples were taken proportionally using a random sampling technique through the lottery method. This approach ensured that every eligible participant had an equal chance of being selected, thus maintaining the integrity of the sampling process. While we acknowledge the value of having a similar proportion of participants from grades 6 to 8, the total number of eligible participants was not uniform across these grades. Therefore, a strict proportionate sampling was not feasible due to variations in enrollment numbers across grades within the selected schools. As long as the total sample size did not exceed the study's capacity to manage and analyze data effectively, a balanced proportion across grade levels was not mandated. A similar approach was used in the non-intervention group as well.

2. **There is no mention of Assent form. Generally for participants aged 12-18 years, an assent from participant and consent from parents is required. Was a written informed assent taken?**

**Response:** Thank you for your questions. We apologize, we forgot to mention written informed assent which was taken before a study and thus it has been corrected in the revised manuscript. Written informed assent from all participants and consent from their parents was obtained in coordination with the school head teacher, ensuring ethical standards and that all participants willingly participated in the study.

**3. There are 6 tables in the Results Section. Plus there is a lot of text. The authors should revise this section to avoid / shorten repeated information present in the both the tables and text.**

**Response:** We appreciate the reviewer's comments. To enhance clarity and conciseness, we have revised this section to eliminate repetitive information. We have ensured that each table is succinctly summarized in the text, avoiding unnecessary repetition of data presented in the tables to ensure that the information is presented clearly and efficiently. We have explained the most significant and key results in the sentence presentation.

**4. Who administered the intervention? What training did that individual receive? Was any pre-intervention rapport built with the participants?**

**Response**: Thank you for your valuable comments. The first author/ principal investigator Saraswati Ghimire (SG) conducted health education intervention. SG received one-day orientation session which was provided by research supervisors on the theoretical foundations and practical aspects of the intervention, including specific methodologies, content development, ethical considerations and participant interaction techniques.

SG possesses a specialized academic background in health promotion and education. This expertise encompasses a deep understanding of effective health educational interventions, including content development, methods of delivery and suitable use of media for disseminating health-related information. Through academic coursework and practical experience, the first author had developed proficiency in implementing health education interventions effectively. Specifically, her familiarity with menstrual hygiene education includes comprehensive knowledge of relevant topics, evidence-based approaches and culturally appropriate methods for delivering educational content through literature review and supervisor guidance. In developing the menstrual hygiene education content, the first author applied their expertise in selecting relevant information and utilizing appropriate media channels. This ensured that the intervention was not only informative but also engaging and accessible to the target audience for example through simulation. The first author's background in health promotion and education played a pivotal role in shaping the intervention’s effectiveness and relevance within the study context.

Pre-intervention rapport building with the participants was deliberately avoided to prevent potential biases or influences on their responses. By skipping rapport building, the research maintained a neutral and unbiased environment, ensuring the integrity and reliability of the data.

**5. As mentioned earlier, the study is relevant. The authors should consider a follow up study of the same group of participants to assess their knowledge and practices at 6 months and 1 year post-intervention to gauze the compliance of the participants to the menstrual hygiene methods included in the intervention.**

**Response:** Thank you for your insightful suggestion regarding a follow-up study to assess participant's knowledge and practices at 6 months and 1-year post-intervention.

The study was limited to a short period due to several potential drawbacks such as participant attrition over time could have result in a reduced and biased sample, while recall bias may cause participants to struggle with accurately remembering past behaviors. Additionally, the Hawthorne Effect might have lead participants to alter their behavior simply because they know they are being studied. External factors, such as social influence, may have impact participant's practices independently of the intervention. Long-term studies are costly and resource-heavy, demanding considerable time and money. Moreover, ethical issues like protecting participant privacy and maintaining ethical practices over time add to the challenges.

Our study focused on the immediate impact of the intervention with focused on using resources efficiently. High-impact interventions through the use of PowerPoint presentations, videos, group discussions mini-lectures, simulations and handout distribution focused on practical aspects that convey powerful messages. This approach aimed to improve both understanding and practice of good menstrual hygiene within a short period of time, avoiding the need for lengthy study. Our study findings showed significant improvement in the knowledge and practice level of adolescent girls on menstrual hygiene after health education intervention which was of short duration.

**We sincerely appreciate the editors and reviewers for their valuable insights into our current submission. We have thoroughly addressed all concerns raised by both the reviewers and the editor, ensuring our revisions align with their expectations. We are optimistic that our article will be published this time, making a significant contribution to the scientific community. We aim to raise awareness of menstrual health education globally, ensuring more people are informed about this crucial topic.**
